# Impact of Servant Leadership on the Work Environment and the Attitudes and Behavior of Nursing Professionals as a Function of Gender: A Systematic Review

**DOI:** 10.1155/jonm/8825138

**Published:** 2025-05-29

**Authors:** Steven Saavedra, Pablo Ruiz-Palomino, Rosa Pérez-Contreras, Juan D. Gonzalez-Sanz

**Affiliations:** ^1^Interdisciplinary Gender Studies Doctoral Program, University of Huelva, Huelva, Spain; ^2^Business Administration Department, University of Castilla-La Mancha, Cuenca, Spain; ^3^Nursing Department, University of Huelva, Huelva, Spain; ^4^Nursing Department, COIDESO Research Center, University of Huelva, Huelva, Spain

**Keywords:** attitudes, gender, nursing, servant leadership, working environment

## Abstract

**Objective:** To evaluate the impact of servant leadership (SL) on the work environment and the attitudes and behaviors of nursing professionals by examining the existing differences according to gender.

**Background:** Leadership styles influence both the attitudes and behavior of people over whom they are exercised and the work climate. SL, a prosocial form of leadership, characterized by the recovery of closeness between the leader and the team members, is one of the most important leadership styles in the nursing field today. Differences in the leadership style developed may occur as a function of gender.

**Evaluation:** A systematic review of the literature was conducted following PRISMA 2020 guidelines. The search spanned from 2000 to 2024 across the databases Web of Science, Scopus, PubMed, Science Direct, and CINAHL. The authors performed the review based on a search syntax, inclusion and exclusion criteria, and the data-extraction process. The synthesis categorized the studies according to their focus on leadership types.

**Key Issues:** The review identified a total of 2140 records, with an additional 49 identified through snowball sampling. After thorough screening, fourteen studies were included in the final review, with an overall sample of 7041 participants, mostly female nurses. SL was positively related to aspects such as nurses' behavior and attitudes, quality leader-nurse relationships, and psychological safety mechanisms, among others.

**Conclusions:** SL has a positive impact on the work climate and attitudes and behaviors of nursing professionals. We did not find significant differences according to gender, as no studies regarding these differences were revealed in the nursing context. Therefore, further research on the impact of SL as a function of gender is essential.

**Implications for Nursing Management:** The application of SL improves the work environment and the attitudes and behaviors of nurses and can therefore improve the quality standards of service offered to patients in hospitals.

## 1. Introduction

There is substantial evidence regarding the influence of work climate on the outcomes of nursing professionals. However, the interaction between the various factors that constitute the work climate remains unclear [1–5]. The COVID-19 pandemic has further accentuated this influence, affecting not only care outcomes but also the health of nurses [6, 7].

The impact of different leadership styles on the work environment is well documented. Specifically, the behavior and leadership skills of healthcare managers can significantly modify the work climate, which is strongly correlated with the outcomes for patients and the satisfaction of healthcare professionals [8–14].

Recently, many authors have been researching change-focused leadership [1, 15, 16]. In the nursing field, this type of leadership seems to help prevent nurses from wanting to leave their job and even the profession and to reduce burnout among nursing professionals [17–20]. Unfortunately, professional settings where this leadership style is stable and enduring are rarely found [4].

Despite advances in the conceptualization of leadership, there are still reasonable doubts about the best way to lead a nursing team. This has led to an increase in studies evaluating the influence of different leadership styles. For example, Wong et al. [21] applied the authentic-leadership model to nursing management; Downey et al. [22] explored the model of informal leaders; Sellgren et al. [13] proposed the development of “super-leadership”; and Avolio et al. [23] and Stansbury [24], respectively, advocated authentic leadership and ethical leadership.

Studies increasingly highlight the positive influence of ethical leadership on the well-being of professionals and the work climate, patient safety, the avoidance of negative situations, and other aspects such as bullying and the poor mental health of nursing professionals [25–31].

Greenleaf's [32, 33] servant leadership (SL) approach includes ethical aspects and has recently been consolidated as a theoretical model for studying leadership in nursing. A central characteristic of this style is the recovery of closeness between the leader and team members, which has been lost with the increase in organizational size and bureaucratization.

Unlike transformational leadership, SL prioritizes the interests of team members over those of the organization and the leader's own interests [34, 35]. This implies an altruistic vocation [36] and an intrinsic motivation towards service [37].

SL, as conceptualized by Greenleaf [32, 33], is a prosocial form of leadership that emphasizes recovering the closeness between the leader and team members, which has been lost as a result of bureaucratization. SL has gained significant support for its promising results in team management and is positively associated with various aspects of the work climate, attitude, and behavior.

SL is positively associated with the unlimited inclusion of followers, customers, and communities [38–40], follower satisfaction, creativity, and engagement [35, 41–47], innovation and teamwork [48, 49], environmental awareness [50–52], organizational performance, and customer satisfaction [40, 53].

These positive associations likely result from characteristics of SL such as authenticity, humility, integrity, compassion, responsibility, courage, altruism, and listening [42, 54]. Servant leaders positively influence those they serve, prioritizing their well-being and personal and professional growth [55–58]. This leadership style has a positive impact on job performance at both individual and team levels [34, 59, 60].

The characteristics that define SL make it more effective than other leadership styles, such as transformational, ethical, and authentic leadership [61]. SL is effective in multiple scenarios, including teaching, as it encourages innovative behavior [62].

The nursing organization should not be oblivious to this. In nursing, SL not only positively impacts patient satisfaction and nursing professionals' job satisfaction [57, 63] but is also key in developing the research capacity of these professionals, fostering values such as collaboration and cooperation [64]. When middle managers practice SL, the work environment is likely to be positively affected, leading to an empowered and motivated workforce. This can enhance outcomes for professionals, patients, and work environments [65, 66]. At higher management levels, SL can be promoted among healthcare supervisors, aligning with Greenleaf's [33] principle that a good servant leader helps followers become servant leaders.

Nurse managers must consider the context (region, culture, and environment) as a set of complexities influencing nurses' satisfaction [45]. Additionally, they must recognize that gender can affect the impact of their leadership style on work climate and followers' attitudes and behaviors. According to Festinger's [67] cognitive dissonance theory, individuals strive to maintain harmony in their attitudes and behaviors. The social role theory [68] suggests that females are more concerned than males with moral, social, and justice issues [69], making female managers and workers more aligned with SL values. Analyzing gender differences in leadership positions could reveal impacts on organizational and attitudinal variables. Recent studies indicate that men still emerge as leaders more often than women [70]. Lemoine and Blum [71] found that 61% of leadership positions were held by men versus 39% by women. Although the gender gap has narrowed [72, 73], it persists [74]. Research on SL's effectiveness when exercised by women could help reduce this gap.

Being perceived as a leader in organizational contexts is associated with higher job performance ratings [75]. Although, on average, gender differences in efficacy are virtually nonexistent [73], and women tend to underestimate themselves as leaders [72], which highlights the importance of studying how women's leadership identity is constructed [76]. The communitarian nature of SL [77] aligns with feminine stereotypes [71, 74], potentially making this leadership style more effective in organizations. SL's impact is expected to vary depending on the gender of leaders and workers [78], with teams oriented towards female gender roles reacting more positively to this leadership style [71].

The aim of this review is to evaluate the impact of SL on the work environment and on nurses' attitudes and behaviors. Through a systematic literature review, we examine the differences in SL's impact based on gender.

## 2. Method

This systematic literature review was conducted according to the guidelines [79], by qualitative synthesis of the literature.  Table 1 shows the participants, intervention, comparison, and outcomes (PICO) criteria used for study inclusion.

### 2.1. Eligibility Criteria

The present review covers studies published from 2000 to 2024. The inclusion criteria were as follows: (1) only studies that were originally published in English and/or Spanish as the authors' native language, (2) study subjects are nurses, (3) only SL is discussed, and (4) PICO criteria must be met. Exclusion criteria are as follows: (1) studies not originally published in English and/or Spanish, (2) studies that cover professionals other than nurses, (3) studies that discuss leadership models other than SL, and (4) studies that do not meet the PICO criteria and/or do not meet the objective of the review.

### 2.2. Sources of Information and Search Strategy

A literature search was conducted from January 2000 to April 2024, using the following sources: *Web of Science*, *Scopus*, *PubMed*, *Science Direct*, and *CINAHL*. The searches were conducted in English and Spanish, and the DeCS descriptors used were as follows: “*attitudes,*” “*behavior,*” “*nurse,*” “*nursing,*” “*gender,*” “*gender role,”* “*role congruity,*” “*work environment,*” and “*working environment,*” together with free text words: “*job response,”* “*servant leadership,*” and “*servant leader.*” The DeCS descriptors used in Spanish were as follows: *“actitudes,” “comportamiento,” “enfermera,” “enfermería,” “género,” “rol de género,” “congruencia de roles,” “entorno de trabajo,” “respuesta del trabajo,” “liderazgo servidor,”* and *“líder servidor.”*

For the searches, the Boolean operators “*and*” and “*or*” were applied, and these were filtered by the title, abstract, and/or keywords to have more exhaustive searches; when no results were found, filters by topic were applied to obtain results. The various searches are listed in Table 2. Furthermore, additional records were identified through manual searches and from the reference lists of the most relevant studies.

### 2.3. Study Selection Process

**Figure 1:**
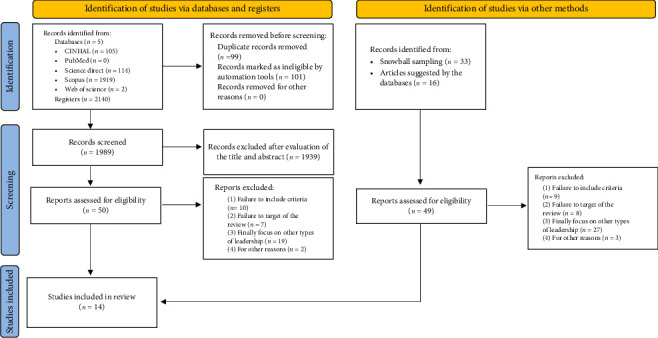
PRISMA (2020) flowchart describing the selection process of the studies included in the systematic review.

The selection process is shown in the flow chart illustrated in Figure 1. The search strategy yielded a total of 2140 records (*CINAHL n* = 105, *PubMed n* = 0, *Science Direct n* = 114, *Scopus n* = 1919, and *Web of Science n* = 2). In addition, a further 49 records were identified, yielding a total of 2189 records. Duplicate studies (*n* = 99) were then removed by reviewers, and other studies were removed (*n* = 101), marked as ineligible by automation tools. The prescreened records (*n* = 1989) were examined in two stages. First, an evaluation by the title and abstract was performed, where 1939 records were eliminated. Second, the remaining selected full-text articles (*n* = 50) were read thoroughly to assess their inclusion in the review. Articles that did not meet all inclusion criteria or did not meet the objective of the review were excluded (*n* = 38). The additionally identified articles (*n* = 49) were also read, and those that did not meet the inclusion criteria or did not respond to the objective of the review were excluded (*n* = 47). Finally, the set of articles included in the present systematic review represented a total of 14 records.

### 2.4. Data Extraction Process and Data List

Data were extracted from the included studies using a data-extraction form for qualitative studies, and the most relevant information from the 14 records included in this review is summarized in Table 3. This table homogeneously compiles all the detailed and relevant information for the analysis, synthesis, and interpretation of the data. Data from the primary sources include author and year, country, study design, participants, sex, sample size, study area, SL assessment, key findings, and article quality.

### 2.5. Risk-of-Bias Assessment

The tool described by López de Argumedo et al. [85] for systematic reviews was used. This tool uses a table (Table 4) to present the assessment of the methodological quality of the study, taking into account the responses to six areas that evaluate the quality of the evidence provided by the included study. The quality of each of the studies is shown in Table 5 with the responses to the different sections of the tool. The analysis revealed that nine studies were of high quality and the remaining five of medium quality. The STROBE statement was also applied (Table 6).

### 2.6. Synthesis of Results

A narrative synthesis of the data was performed by systematically extracting general data, the evaluation performed, and key findings in reference to the objective of the review from each study, as detailed in Table 3. The quality of each study was evaluated using the criteria outlined by Argumedo et al. [85], with results presented in Tables 4 and 5. The extracted data were organized to identify patterns, similarities, and differences across the studies, and detailed descriptions of each study were provided to understand the scope and quality of the evidence. The findings from the individual studies were then integrated into a coherent narrative, summarizing the evidence, explaining how the studies relate to each other, and drawing conclusions based on the collective data.

## 3. Results

### 3.1. Selection of Studies

The study selection process is described in the PRISMA (2020) flow chart (Figure 1). Initially, 2140 records were identified from CINHAL, Science Direct, Scopus, and Web of Science. An additional 49 records were identified through snowball sampling and articles suggested by the databases. After removing 99 duplicate records and 101 records marked as ineligible by automation tools, 1989 records were screened based on their titles and abstracts. Of these, 1939 records were excluded for not meeting the inclusion criteria.

The remaining 50 reports were assessed for eligibility through full-text evaluation. During this phase, 36 reports were excluded for various reasons, such as failure to meet inclusion criteria, not targeting the review's focus, or focusing on other types of leadership. Ultimately, 14 studies were selected for inclusion in the systematic review.

### 3.2. Characteristics of the Studies

Table 3 provides a brief synthesis of the 14 studies reviewed, where the articles are ordered alphabetically. The 14 included studies were published over a nine-year period (2014–2023). Demographically, the studies covered countries in North America (United States), Asia (China, India, Malaysia, and Pakistan), Europe (Sweden and the Netherlands), and Turkey, which has territory in Europe and Asia. Sample sizes ranged from 14 to 1604 participants, bringing the overall sample to a total of 7041 participants. In terms of gender, except for the study by Ahmad et al. [86], all studies were mostly or exclusively composed of women. All samples were composed of nursing staff, except for the study by Ahmad et al. [86] and that by Omanwar and Agrawal [18], which did not specify the type of staff, including whether they were other healthcare professionals. With respect to the area of study, all studies were conducted in the hospital setting, apart from Westbrook et al. [94], which also included other healthcare settings.

Most studies were observational studies (*n* = 12), with convenience sampling, studies with experimental design (*n* = 1), and studies with qualitative design (*n* = 1). As for the SL assessment tool, the studies used the 30-item SL scale by van Dierendonck and Nuijten [83, 96] (*n* = 3), the seven-item SL scale by Liden et al. [40, 55] (*n* = 4), the 28-item SL scale by Liden et al. [39] (*n* = 3), Ehrhart's [81] 14-item SL scale (*n* = 1), Qiu and Dooley's [82] 24-item SL scale (*n* = 1), and Patterso's [80] 71-item SL scale (*n* = 1).

### 3.3. Findings on the Impact of SL in Nursing on the Work Environment and on the Attitudes and Behaviors of Nursing Professionals

The included studies assessed the impact of SL in nursing on the work environment through enhancing team effectiveness, trust in the leader, collaboration between professionals, quality of the relationships between the leader and nurses, the work climate, and the safety of the hospital environment, as well as its quality standards, among other factors. In addition, the included studies assessed the impact of SL on nurses' attitudes and behaviors, with findings including its impact on nurses' psychological empowerment, creativity, psychological safety, work behavior and commitment, job burnout, patient satisfaction, job satisfaction, intention to stay in the job and the organization, and identification with the organization.

### 3.4. Findings on Differences in the Impact of SL on the Work Environment and on the Attitudes and Behavior of Nursing Professionals According to Gender

No included studies compared the data according to the sex of the participants, whether or not they were workers or leaders.

## 4. Discussion

We will now describe the impact of SL in nursing on the work environment and on the attitudes and behaviors of nursing professionals. It is important to note that we will do so by studying the existing differences according to gender, although this could not be performed adequately because, as mentioned above, no individual study compared the data according to the participants' gender.

### 4.1. Impact of SL on the Work Environment and Nurses' Attitudes and Behaviors

The impact of SL on the work environment is influenced by demographic, cultural, and political factors, among others. Given this influence, choosing the most appropriate leadership style in each case is challenging. However, SL appears to be effective as described below.

Three studies from the United States were included. An observational study showed that SL improved nurses' job satisfaction and, consequently, patient satisfaction [57]. The same study also found that SL enhanced nurses' creativity, collaboration, and helping behavior. Additionally, formalized organizational structures enhanced the association between SL and patient satisfaction through improved job satisfaction and creativity. Specchia et al. [66] found similar results, showing a strong relationship between SL and job satisfaction, leading to better care outcomes. Another observational study [94] found that SL decreased work environment stressors and burnout, indirectly improving job satisfaction. However, SL did not directly reduce job-change intentions. Malak et al. [84] concluded that SL by managers modified nurses' behavior, improving hospital safety and quality standards.

Two European studies were included. One conducted in Sweden [87] showed that SL positively influenced the relationship between supervisors and nurses, likely improving care quality. This aligns with previous studies [63, 97], which found that SL fosters professional growth and better healthcare delivery through teamwork, shared decision-making, and ethical behavior. Another study from the Netherlands [35] analyzed SL mechanisms and their effect on work engagement. SL improved nurses' perception of their managers' effectiveness, positively influencing organizational commitment and work engagement. Decuypere and Schaufeli [98] found similar results, indicating that SL positively affects work engagement by providing moral-manager support, modeling behavior, and fostering positive exchanges. Parris and Peachey [99] showed that SL improves trust, leader effectiveness, collaboration, and work climate.

Five observational studies from Pakistan were included [86, 88, 90, 93, 95]. Ahmad et al. [86] found that SL reduces the negative impact of peer mistreatment on burnout, improving organizational commitment. SL fosters a favorable work environment for commitment and innovation, positively impacting service attitude and innovative behaviors. During the COVID-19 pandemic, Ma et al. [90] found that SL created a psychologically safe work environment, reducing burnout. Saleem et al. [93] indicated that SL increases trust in leaders, benefiting hospital performance and care quality. Yasir and Jan [95] concluded that SL reduces negative work behaviors and perceptions of an unfair organizational climate, contributing to organizational effectiveness. Ul Hassan et al. [88] found that SL reduces turnover intention, even in the presence of workplace bullying. Omanwar and Agrawal [18] found that SL increases identification with the organization and reduces job change intentions.

In Turkey, one study [89] found that SL positively relates to nurses' innovative behavior, enhancing job performance. SL likely enhances the positive relationship between innovative behavior and job performance, fostering proactive behaviors and creative attitudes.

In China, Qiu and Zhang [92] found that SL buffered the negative effect of uncivil behavior on nurses' psychological and emotional safety during the pandemic. Ma et al. [90] and Saleem et al. [93] found similar results, indicating that SL positively impacts psychological safety, reduces burnout, and increases trust in leaders. Qiu and Zhang [92] concluded that SL reduces the negative effects of organizational incivility on psychological distress by creating an ethical work climate conducive to emotional healing and resilience.

### 4.2. Differences in the Impact of SL on the Work Environment and Nurses' Attitudes and Behaviors as a Function of the Gender Variable

As mentioned in the introduction, gender differences still exist in managerial positions [72–74], with mostly men occupying these positions despite women identifying better with SL characteristics: 61% of men versus 39% of women managers [71].

None of the included studies specifically examines the gender variable or discusses results based on participants' gender or sex, providing only demographic data on sample representation. All samples are predominantly female, apart from Ahmad et al. [86] (see Table 3). However, Parris and Peachey [99] compared this variable and found differences in SL style usage between male and female workers. Female servant leaders identified more with aspects of SL, such as consensus building, fostering self-esteem, and engaging in healing relationships, consistent with Lemoine and Blum [71] and their claims about female stereotypes.

The study from Turkey [89] describes how the SL score, female gender, and institution (university hospital) significantly affect nurses' job performance. However, the sample of male nurses is insignificant, and the high number of female nurses might have influenced these results.

Data from our review, along with other research [71, 99], are interesting regarding gender and its influence on SL. Nonetheless, because of the scarcity of data, results cannot be extrapolated to the nursing sector. Future studies should explicitly evaluate the role of gender in SL's impact on work climate and nursing professionals' attitudes and behaviors.

### 4.3. Limitations

Research on SL has increased in recent years, probably thanks to studies showing that this leadership style is more effective than other highly effective leadership styles (transformational leadership, ethical leadership, and authentic leadership). However, research on this leadership style remains scarce, especially in nursing. This has been a limitation in the design of this study, given the small number of studies that met the requirements we had set out to address (i.e., possible differences in the impact of SL as a function of gender, as we were able to include only one article dealing with this issue). Thus, more research is needed that takes gender into account as a factor, and more research is also needed that reduces the problems of clarity that exist in the current literature in relation to the building, measurement, and design of SL, which may have produced conceptual overlaps with other leadership styles [34].

An important limitation was the lack of literature on the influence of gender on the impact of SL in nursing. Only the review by Parris and Peachey [99] analyzed this variable, but as it was a literature review and included several sectors, it was discarded from our research. Therefore, this variable (i.e., gender) needs to be taken into account in future research. Although the trend is changing and there are more and more female leaders, there is still a notable gap with the male gender, and further research on the positive impact that the female gender variable is likely to have could help hospital managements to reduce this gender gap.

A final limitation is the generalizability of the results of the included studies (external validity), since as a result of the heterogeneity of existing cultures and the paucity of samples, it is difficult to extrapolate the results. Thus, it is necessary to expand the area of study and the samples in future research to obtain data that can be generalized to the rest of the population.

### 4.4. Implications for Managerial Nursing Practice and Future Research

The results obtained are of interest for healthcare practice, as they reveal that the adoption of an appropriate leadership style can contribute to creating a better work environment and increase nurses' well-being and positive behaviors and attitudes. In fact, this style of leadership is likely to be of relevance in critical situations, as for example occurred during the COVID-19 pandemic, which required leaders with adequate competencies to cope with this type of situation.

Although the findings in the present review permit the claim that SL is an effective leadership style that helps to improve the work environment and nurses' well-being and to encourage valuable attitudes and behaviors, more research is needed to affirm the external validity of these findings. This systematic review can serve as a basis for future research in the nursing field. In addition, there is a need to investigate the influence of gender on the outcomes that a servant leader can provide in terms of work climate, attitudes, and the behaviors of nursing professionals. Given the paucity of data and studies in the nursing field on these factors and given that gaps between men and women in management continue to exist, future research on the topic should focus on assessing whether the gender variable has a say in the positive impact a servant leader has on the work climate and on the attitudes and behaviors of nurses.

## 5. Conclusions

SL has a positive influence on the work environment of nursing professionals and on multiple attitudinal and behavioral variables, including the quality of care they provide. The present review contemplates how SL has a positive impact on the work environment (by reducing stressors and shaping a fair climate) as well as on the psychological empowerment of nurses, their job satisfaction, and their innovative behavior. Our systematic review also reveals how SL helps decrease emotional exhaustion of nurses in critical situations and how it improves the quality of the relationship nurses have with their superiors. Although research in SL is expanding, further research is needed. Research in nursing is scarce, and the external validity of the studies of the last decade is inconsistent because of the limited geographical range of the studies and the limited samples analyzed. In addition, research on gender differences in SL is virtually nonexistent, so it is critical to investigate in future research the role of gender on leadership effectiveness, particularly on the impact of supervisors' SL in a nursing context.

## Figures and Tables

**Table 1:** PICO criteria for inclusion of studies.

Parameter	Criterion
Participants	Nurses, intermediate positions (supervisors), and senior positions (directors)
Intervention	—
Comparison	Between men and women (gender variable)
Outcomes	Impact of servant leadership in nursing on the work environment and on the attitudes and behaviors of nursing professionals

**Table 2:** Search of the literature.

Source	Search	Total records
CINAHL	1. (“nursing” OR “nurse”) AND (“servant leadership” OR “servant leader”) AND (“attitudes” OR “job response” OR “behavior”) AND (“gender role” OR “gender” OR “role congruity”) (all text) [68]	105
	2. (“nursing” OR “nurse”) AND (“servant leadership” OR “servant leader”) AND (“work environment” OR “working environment”) AND (“gender role” OR “gender” OR “role congruity”) (all text) [37]	
	^∗^(“servant leadership” OR “servant leader”) AND (“attitudes” OR “job response” OR “behavior”) AND (“gender role” OR “gender” OR “role congruity”) (all text) [99]	
	^∗^(“servant leadership” OR “servant leader”) AND (“work environment” OR “working environment”) AND (“gender role” OR “gender” OR “role congruity”) (all text) [54]	

PubMed	1. (“nursing” OR “nurse”) AND (“servant leadership” OR “servant leader”) AND (“attitudes” OR “job response” OR “behavior”) AND (“gender role” OR “gender” OR “role congruity”) [0]	0
	2. (“nursing” OR “nurse”) AND (“servant leadership” OR “servant leader”) AND (“work environment” OR “working environment”) AND (“gender role” OR “gender” OR “role congruity”) [0]	
	^∗^(“servant leadership” OR “servant leader”) AND (“attitudes” OR “job response” OR “behavior”) AND (“gender role” OR “gender” OR “role congruity”) [title] [0]	
	^∗^(“servant leadership” OR “servant leader”) AND (“work environment” OR “working environment”) AND (“gender role” OR “gender” OR “role congruity”) [0]	

Science Direct	1. (“nursing” OR “nurse”) AND (“servant leadership” OR “servant leader”) AND (“attitudes” OR “job response” OR “behavior”) AND (“gender role” OR “gender” OR “role congruity”) (find articles with these terms) [65]	114
	2. (“nursing” OR “nurse”) AND (“servant leadership” OR “servant leader”) AND (“work environment” OR “working environment”) AND (“gender role” OR “gender” OR “role congruity”) (find articles with these terms) [49]	
	^∗^(“servant leadership” OR “servant leader”) AND (“attitudes” OR “job response” OR “behavior”) AND (“gender role” OR “gender” OR “role congruity”) (find articles with these terms) [493]	
	^∗^(“servant leadership” OR “servant leader”) AND (“work environment” OR “working environment”) AND (“gender role” OR “gender” OR “role congruity”) (find articles with these terms) [211]	

Scopus	1. (“nursing” OR “nurse”) AND (“servant leadership” OR “servant leader”) AND (“attitudes” OR “job response” OR “behavior”) AND (“gender role” OR “gender” OR “role congruity”) (all fields) [1480]	1919
	2. (“nursing” OR “nurse”) AND (“servant leadership” OR “servant leader”) AND (“work environment” OR “working environment”) AND (“gender role” OR “gender” OR “role congruity”) (all fields) [439]	
	^∗^(“servant leadership” OR “servant leader”) AND (“attitudes” OR “job response” OR “behavior”) AND (“gender role” OR “gender” OR “role congruity”) (all fields) [4272]	
	^∗^(“servant leadership” OR “servant leader”) AND (“work environment” OR “working environment”) AND (“gender role” OR “gender” OR “role congruity”) (all fields) [828]	

Web of Science	1. (“nursing” OR “nurse”) AND (“servant leadership” OR “servant leader”) AND (“attitudes” OR “job response” OR “behavior”) AND (“gender role” OR “gender” OR “role congruity”) (topic) [2]	2
	2. (“nursing” OR “nurse”) AND (“servant leadership” OR “servant leader”) AND (“work environment” OR “working environment”) AND (“gender role” OR “gender” OR “role congruity”) (topic) [0]	
	^∗^(“servant leadership” OR “servant leader”) AND (“attitudes” OR “job response” OR “behavior”) AND (“gender role” OR “gender” OR “role congruity”) (topic) [109]	
	^∗^(“servant leadership” OR “servant leader”) AND (“work environment” OR “working environment”) AND (“gender role” OR “gender” OR “role congruity”) (topic) [5]	

**Table 3:** Synthesis of the studies included in the systematic review of the literature.

Author (year)	Country	Design	Participants	Gender	Sample size	Unit of analysis	Servant leadership measurement	Key findings	Quality
Ahmad et al. (2021)	Pakistan	Cross-sectional observational	Health professionals (unspecified)	43% female/57% male	431	Hospital	Liden et al.'s [55] 7-item scale	A SL by managers is critical to fostering employee innovative behavior and may be a working climate aspect that generates service and helping behavior in the workplace	Medium
Hanse et al. (2016)	Sweden	Cross-sectional observational	Health professionals (83% nurses)	82% female/18% male	240	Hospital	van Dierendonck and Nuijten's 30-item scale [37]	SL positively influences the quality of the relationship between the leader and the nurses, measured through four key aspects: “affection,” “loyalty,” “contribution,” “professional respect,” and “professional respect”	High
Hassan et al. (2021)	Pakistan	Cross-sectional observational	Nurses	100% female	285	Hospital	7-item Liden et al.'s [55] scale	SL acts as a buffer against the negative effects of nurses' poor job well-being (which is previously shaped by workplace bullying) on nurses' turnover intention. In addition, SL was observed to directly reduce nurses' turnover intention	High
Kül and Sönmez (2021)	Turkey	Cross-sectional observational	Nurses	91% female/9% male	885	Hospital	Patterson's 71 ítem scale [80]	SL was positively related to innovative behavior and job performance of nurses, as well as acting as a reinforcer of the positive relationship between innovative behavior and job performance of these professionals	Medium
Ma et al. (2021)	Pakistan	Cross-sectional observational	Nurses	94% female/6% male	443	Hospital	Liden et al.'s [55] 7-item scale	Leaders who developed SL generated a psychologically safe work environment, which increased nurses' psychological safety during the COVID-19 pandemic, significantly reducing their burnout	Medium
Malak et al. (2022)	United States	Qualitative with interview	Nursing managers	86% female/7% male^∗^	14	Hospital	—	The SL of hospital nurse managers promotes the emergence in nurses of certain behaviors that improve the safety of the hospital environment and their overall level of quality and performance	High
Mostafa et al. (2023)	Malaysia	Cross-sectional observational study	Nurses	93% female/7% male	345	Hospital	Liden et al.'s [39] 28-item scale	SL helps to reduce the negative consequences of coworker mistreatment on the emotional well-being of nursing professionals and, through this, reduces the negative impact this mistreatment has on the commitment of these professionals to the organization	Medium
Neubert et al. (2016)	United States	Cross-sectional observational study	Nurses and Nursing managers	91% female/9% male	1590	Hospital	Ehrhart's [81] 14-item scale	SL improves nurses' job satisfaction and, through it, patient satisfaction. In addition, SL increases nurses' creativity and helping behavior. In addition, it was observed that more formal organizational structures that clearly state the role of workers in the organization enhance the positive effect of SL	High
Omanwar and Agrawal (2022)	India	Cross-sectional observational study	Health professionals (unspecified)	73% female/27% male	266	Hospital	Liden et al.'s [39] 28-item scale	SL has a positive relationship with employees' organizational identification and reduces their turnover intention	Medium
Qiu and Zhang (2022)	China	Cross-sectional observational study	Nurses	91% female/9% male	1604	Hospital	Qiu and Dooley's [82] 24-item scale	SL has a buffering effect on nurses' psychological distress stemming from the negative impact of a work environment that promotes incivility. Thus, this study demonstrates that a servant leader, through emotional healing, can help employees feel good even when they are subjected to uncivil behaviors	High
Saleem et al. (2022)	Pakistan	Cross-sectional observational study	Nurses	100% female	339	Hospital	Liden et al.'s [39] 28-item scale	SL instills leader trust in leaders among nurses, thereby indirectly increasing hospital performance, and does so more strongly when nurses are psychologically empowered. Interestingly, a correlational analysis revealed that SL may also be behind the greater psychological empowerment of nurses	High
van Dierendonck et al. (2014)	The Netherlands	Experimental study	Nurses and physicians	75% female/25% male	200	Hospital	van Dierendonck and Nuijten's [37] 30-item scale	SL is mainly characterized by contributing to the satisfaction of the psychological needs of healthcare professionals (nurses and physicians) and, through this, improving their work engagement. In addition, SL is also shown to be effective leadership, all of which improves the organizational and work commitment of healthcare professionals and also their work engagement	High
Westbrook et al. (2022)	United States	Cross-sectional observational	Nurses	83% female/17% male	248	Hospital (56%) y otros lugares	van Dierendonck and Nuijten's [83] 30-item scale	SL directly decreases nurses' stressors and burnout and through these factors has an indirect positive influence on job satisfaction. This triad may positively impact nurses' individual performance. However, SL does not directly decrease job turnover intentions. It would do so through decreasing nurses' work-environment stressors and burnout and thereby increasing their job satisfaction	High
Yasir and Jan (2023)	Pakistan	Cross-sectional observational	Nurses	62% female/38% male	201	Hospital	Liden et al.'s [40] 7-item scale	SL modifies nurses' behavior by directly reducing workplace deviance behavior and also by improving the perception of a fair work climate. Thus, SL contributes positively to the organizational effectiveness of any organization	High

^∗^The remaining 7% did not specify the gender (Malak et al.'s [84] study).

**Table 4:** Evaluation of the quality of each study [85].

Research question: Is the study based on a clearly defined research question?	Yes	No	Partially	No information
Method: Did the study method minimize bias?	Yes	No	Partially	No information
Results: Are the results correctly synthesized and described?	Yes	No	Partially	No information
Conclusions: Are the conclusions justified?	Yes	No	Partially	No information
Conflict of interest: Is the existence or absence of conflict of interest well described?	Yes	No	Partially	No information
External validity: Are the results of the study generalizable to the population and context of interest?	Yes	No	Partially	No information

	**Method YES**	**Method PARTIAL**	**Method NO**	

Majority of other criteria YES	High quality	Medium quality	Low quality	
Majority of other criteria PARTIALLY	Medium quality	Medium quality	Low quality	
Majority of other criteria NO	Low quality	Low quality	Low quality	

**Table 5:** Evaluation of the quality of each study.

Reference	Research question	Methods	Results	Conclusions	Conflict of interest	External validity	Quality
Ahmad et al. [86]	Yes	Partially	Yes	Yes	Yes	No	Medium
Hanse et al. [87]	Yes	Yes	Yes	Yes	Yes	Partially	High
Hassan et al. [88]	Yes	Yes	Yes	Yes	No	No	High
Kül and Sönmez [89]	Yes	Partially	Yes	Yes	No	Partially	Medium
Ma et al. [90]	Yes	Partially	Yes	Partially	Yes	No	Medium
Malak et al. [84]	Yes	Yes	Yes	Yes	Yes	Partially	High
Mostafa et al. [91]	Yes	Partially	Yes	Yes	No	Partially	Medium
Neubert et al. [57]	Yes	Yes	Yes	Yes	No	Partially	High
Omanwar and Agrawal [18]	Yes	Partially	Yes	Yes	No	No	Medium
Qiu and Zhang [92]	Yes	Yes	Yes	Yes	Yes	Partially	High
Saleem et al. [93]	Yes	Yes	Yes	Yes	Yes	Partially	High
van Dierendonck et al. [35]	Yes	Yes	Yes	Yes	Yes	Partially	High
Westbrook et al. [94]	Yes	Yes	Yes	Yes	Yes	Partially	High
Yasir and Jan [95]	Yes	Yes	Yes	Yes	Yes	Partially	High

**Table 6:** STROBE statement.

STROBE statement	Total points included in each study
Ahmad et al. [86]	14
Hanse et al. [87]	18
Hassan et al. [88]	16
Kül and Sönmez [89]	14
Ma et al. [90]	14
Malak et al. [84]	18
Mostafa et al. [91]	16
Neubert et al. [57]	17
Omanwar and Agrawal [18]	14
Qiu and Zhang [92]	18
Saleem et al. [93]	18
van Dierendonck et al. [35]	17
Westbrook et al. [94]	18
Yasir and Jan [95]	17

*Note:* The declaration comprises a total of 22 points.

## Data Availability

The data that support the findings of this study are available from the corresponding author upon reasonable request.
